# Design of a Structure for Optimized Optical Performance of a Full Colored Organic Light-Emitting Diode on a Parameter Space Map

**DOI:** 10.3390/polym14030585

**Published:** 2022-01-31

**Authors:** Chang-Hee Lee, Ju-Hyeok Choi, Seo-Yong Hyun, Ji-Ho Baek, Bongsoon Kang, Gi-Dong Lee

**Affiliations:** 1 Department of Electronics Engineering, Dong-A University, Busan 49315, Korea; lchanghee62@gmail.com (C.-H.L.); pps7755@naver.com (J.-H.C.); bongsoon@dau.ac.kr (B.K.); 2 P&H Tech Co., Ltd., Seoul 17015, Korea; simon.hyun@phtech.co.kr; 3 LG Display Co., Ltd., Seoul 07336, Korea; bjhkhj@lgdisplay.com

**Keywords:** OLED, cavity effect, parameter space map, organic films, inorganic films, optical performance

## Abstract

In general, optical properties of a top-emitting organic light-emitting diode (OLED) are dependent on the cavity effect of the OLED structure. Therefore, the optical path length of the many thin solid films in the OLED, which is strongly affected by the refractive index and thickness of each material, controls the cavity effect of the cell. In previous research, a parameter space method for optimizing the inorganic layer thickness of a red OLED structure was introduced to achieve the required bandwidth and peak wavelength. This is a simple method with high accuracy and can also be applied to red, green, and blue OLED structures. To design an OLED cell with a practical approach, however, the RGB OLED device requires the thickness of each inorganic layer and organic layer in all three R, G, and B OLED structures to be same. In this study, we applied the parameter space method to an RGB OLED device to find out and optimize the thickness of three inorganic parameters: Indium Tin Oxide (ITO), cathode, and capping layer (CPL) using the finite-difference time-domain (FDTD) method. The parameters ITO, cathode, and CPL were scanned from 18 to 21 nm, 5 to 100 nm, and 10 to 200 nm, respectively. The peak wavelength and bandwidth lines of the three spectral colors were placed on a map of the three inorganic layer thickness parameters to find the optimized points that can provide the desired optical characteristics with the same film thickness in the cell.

## 1. Introduction

In recent years, organic light-emitting diodes (OLEDs) have been actively studied as high-efficiency and eco-friendly light sources based on organic materials; they are advanced light sources with the spectral distribution closest to natural light [1,2,3,4,5]. White light from an OLED can normally be generated by composing three primary colors: red, green, and blue (RGB) [6,7,8,9]. In the application of OLED for display devices, there are two structures for the light propagating method from the OLED panel, which emit to top and bottom directions from the panels [10]. In particular, the top emitting method can be applied to small OLED panels that require high resolution properties because the method is not affected by the area of a thin film transistor (TFT). Instead, the top emitting structure normally uses both the mirror and half-mirror structure for the resonance in the visible wavelength ranges, so the cavity effect of the many organic and inorganic films used in OLED structures should be considered for their high optical properties [11,12,13]. To obtain the required bandwidth and peak wavelength depending on the color of each structure, we normally change the optical length by changing the thickness of each layer in each RGB in all structures [14]. Therefore, the optical characteristics cannot be obtained if the layer thickness in each RGB structure is not the same.

Several researchers have used a numerical approach to perform the spectrum at visible wavelengths and calculate the full width at half-maximum (FWHM) and central wavelength in the RGB structure in each situation of changing layer thickness or optical constant index [15,16,17,18,19]. Therefore, the thickness can be optimized based on the required optical characteristics. However, as such studies deal with the optical thickness of the organic layer, the optical characteristics of the device may be altered by changes in the optical length if the organic layer deteriorates the optical characteristics. Therefore, this method is very complex in computation, and it is difficult to observe bandwidth and peak wavelength changes with changes in layer thickness.

In this study, a parameter space method was used to overcome this drawback [20]. The parameter space method was applied in red, green, and blue structures to optimize the layer thickness with simulations in Lumerical FDTD Solution software (Lumerical Inc., Vancouver, BC, Canada), a simulator based on the finite-difference time-domain (FDTD) method [21,22]. This is a simple method with high accuracy, even in anisotropic materials, and easily presents the relationship between parameters and output results. In this study, we applied the parameter space method to three desired wavelengths: red (618 nm), green (534 nm), and blue (460 nm) in a white top emitting OLED to easily optimize the thickness of the parameters of inorganic films, with the thickness of the organic layers in each RGB structure being equally optimized. First, we applied the parameter space method in each structure to find the range of thickness of the inorganic layers with the required peak wavelength and bandwidth. Then, we presented the peak wavelength and bandwidth of all three colors on a map and found the matched point of these optical characteristics with closest values. Therefore, the optimized thickness of the layers for all three structures can be obtained with minimal error. Finally, the results of the optimized thickness in all three structures were compared with the experimental results to verify the calculations.

## 2. Method

**Figure 1:**
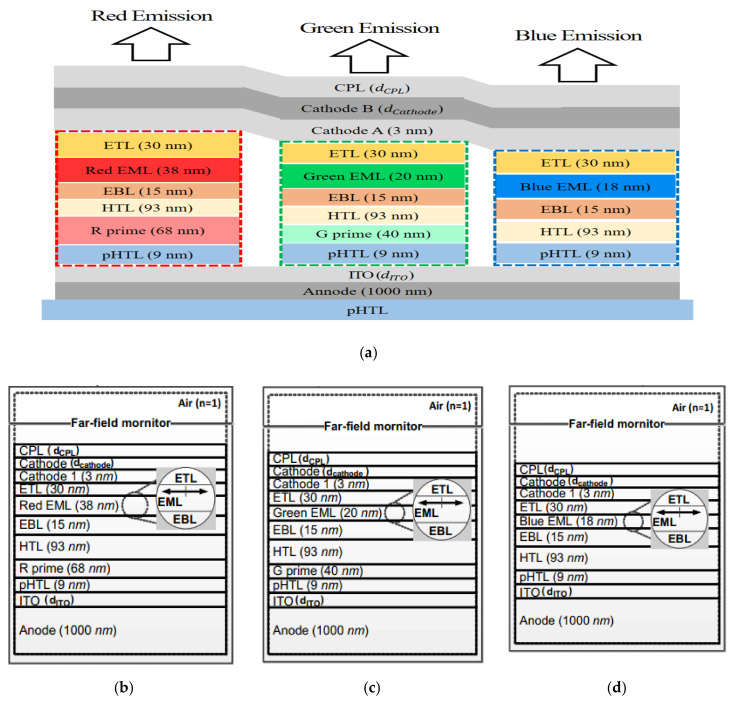
(**a**) The structures of red, green, and blue organic light-emitting diode (OLED) and simulation of (**b**) Red OLED, (**c**) Green OLED, and (**d**) Blue OLED.

The structures of the red, green, and blue OLEDs are shown in Figure 1a. Basically, all three OLED structures have the same layers: capping layer (CPL), cathode, cathode 1, electron transport layer (ETL), electron blocking layer (EBL), hole transport layer (HTL), p-doped hole transport layer (p-HTL), indium tin oxide (ITO), and anode. Above all, owing to anisotropy materials, uniaxial data for HTL, Rprime, Gprime, and p-HTL were applied for precise calculations. The optical axis of the uniaxial layers is along the normal axis with reference to the substrate. The different layers of each structure were an emitting layer (EML) and prime layers with different materials and thicknesses. The materials of the red and green emitting layers were a phosphorescence material, and that of the blue material was a fluorescence material. The optical characteristics at 534 nm wavelength of the isotropic and uniaxial materials used in this study are summarized in Table 1 and Table 2.

**Figure 2:**
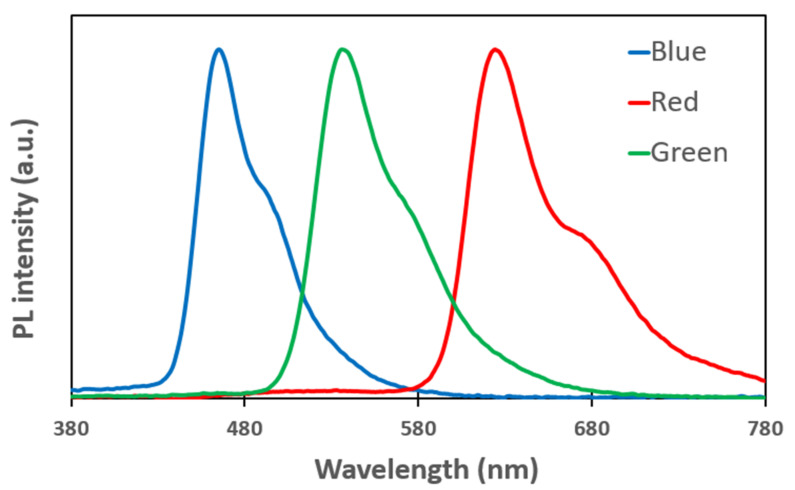
Measured the photoluminescence spectrum of the used R, G, and B emitting layers (EML).

Our study expected a bandwidth of 24 nm in each red (λ = 618 nm), green (λ = 534 nm), and of 18 nm in blue (λ = 460 nm) wavelength by optimizing the optical path of the OLED cell. The spectrum bandwidth is defined at a wavelength range that is the half-intensity of the maximum intensity in the wavelength range, and the wavelength position of the maximum intensity becomes the dominant wavelength. Figure 2 shows the photoluminescence spectrum of the used R, G, and B emitting layers (EML).

**Figure 3:**
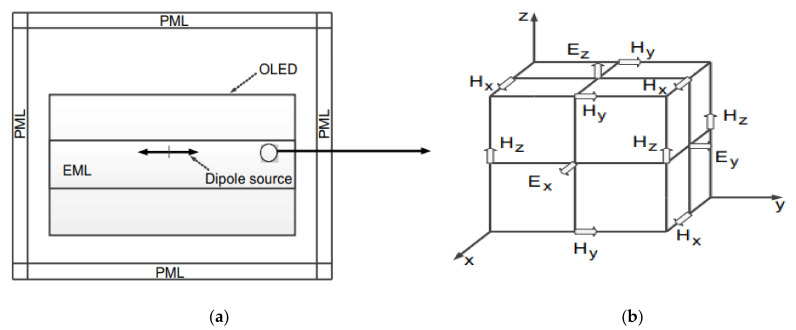
(**a**) Simulation of OLED with finite-difference time-domain (FDTD) method and (**b**) Yee cell in simulation.

For precise calculation in this study, the finite-difference time-domain (FDTD) method was used to calculate optical performance. The FDTD method is a computational electromagnetics analysis technique that solves Maxwell’s equations in a sequence of events in the time domain [22]. The space is discretized to the cubic Yee cell with electric and magnetic field vectors at the center and edge of the cell, as shown in Figure 3b. The time is quantized into steps to represent the travel time between cells.

From the differential equation of Maxwell’s Equation (1) given below, we can see that the change in the E-field over time depends on the change in the H-field across space [23].

(1) $$H\left(t+∆t/2\right)=H\left(t-∆t/2\right)-\frac{∆t}{\mu }\left(\nabla \times E\left(t\right)\right),$$

this creates a basic FDTD time-stepping relationship in which the updated value of the E-field at any point in space depends on the stored value of the E-field and the numerical curl of the distribution of the H-field.

(2) $$E\left(t+∆t\right)=E\left(t\right)-\frac{∆t}{\varepsilon }\left[\nabla \times H\left(t+∆t/2\right)\right],$$

In the Equation (2), the H-field is also time-stepped in a similar way, where the updated value of the H-field over time at any point in space depends on the stored value of the H-field and the numerical curl of the distribution of the E-field. Therefore, the electric and magnetic fields in the cells are updated for each time step [24].

The FDTD calculations provide numerous advantages in terms of electromagnetic numerical calculations [25]: simple implementation, wide-band frequency simulation, and visualization of electromagnetic fields [26]. A special advantage is that optical anisotropy problems due to layer inhomogeneity do not need to be considered when the incidence is oblique. Thus, simple and accurate results can be expected for optical properties.

The light emitted from the emitting layer (EML) was modeled using an isotropic dipole source that is oriented along the x, y, and z axes in the simulation [27]. To obtain the emitted isotropic light, we can simply sum the results incoherently. This means that ${\left|\vec{E}\right|}^{2}=\frac{1}{3}\left\{{\left|{\vec{E}}_{x}\right|}^{2}+{\left|{\vec{E}}_{y}\right|}^{2}+{\left|{\vec{E}}_{z}\right|}^{2}\right\}$. Simulation domain sides were used as perfect match layers to minimize reflection errors [28] and eliminate numerical instability without unnecessarily increasing the simulation time, as shown in Figure 3a [25]. Each structure was simulated separately in an area of 10 μm × 10 μm, as shown in Figure 1b–d. In this paper, parameter layers for optimizing the optical characteristics of the OLED were determined with inorganic layers, which are a CPL, cathode, and ITO layer in the structure because change in the optical thickness of the organic layers would affect the even electrical property of the OLED. In addition, in order to find out higher applicable structure of the cell, we applied same optical thickness of three inorganic layers in all three R, G, and B OLED structures. To measure the intensity of the emitted light for each R, G, and B structure, a far-field monitor was placed on the capping layer.

These results were inserted into the MATLAB program to calculate the peak of wavelength and bandwidth of the emitted spectra. Then, we could make a map that can provide the calculated bandwidth and the peak wavelength peak as functions of the optical thickness of the three parameter layers, so that we could finally find out the optimized thicknesses of CPL, cathode, and ITO for all three R, G, and B color OLED cells.

## 3. Results

The parameter space method was applied to calculate the optimized point including the desired peak wavelength and bandwidth. As mentioned before, we calculated the optimized optical path length *n* (refractive index) × *d* (thickness) as functions of thickness of the inorganic layers the CPL, the Cathode, and the ITO because change in the organic films can induce the deterioration of the electro-optical properties. The thickness ranges of ITO, Cathode, and CPL were set in the ranges of 13–22 nm, 5–100 nm, and 10–200 nm, respectively [20].

**Figure 4:**
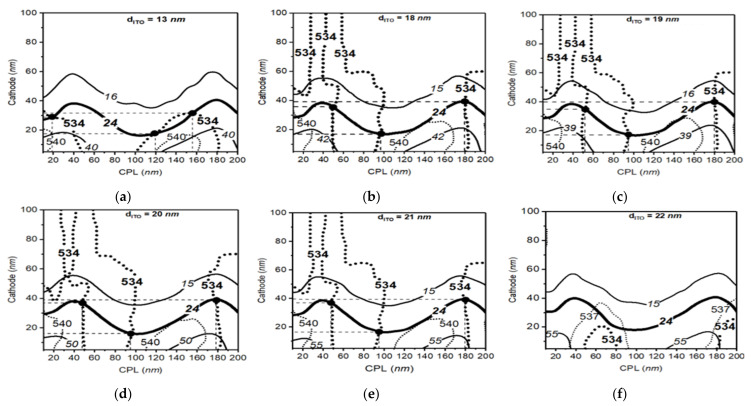
The parameter space result of green structure with indium tin oxide (ITO) thickness from 13 to 22 nm. Solid lines and dotted lines in the figure show the calculated output lines in each optical thickness of the capping layer (CPL) and cathode layer. Bold lines in the figure represent the desired optical conditions with the peak wavelength of 534 nm and bandwidth of 24 nm for the green OLED (**a**–**f**).

In the case of the green OLED cell, our calculation on the parameter space map had a goal of the peak wavelength of 534 nm and bandwidth of 24 nm, and the parameter space map show the optimized points that can provide the target optical performances, as shown in Figure 4. In Figure 4a–f, we obtain several optimized conditions for the green OLED by changing the thickness of the inorganic layers. In Figure 4a, for example, when the ITO thickness was 13 nm, the optimized CPL and cathode thickness showing 534 nm peak wavelength and 24 nm bandwidth were 20 nm and 30 nm, 120 nm and 17 nm, and 158 nm and 31 nm, respectively. In Figure 4b–e, we also can see three optimized points on the map within the given ranges, similar to the results of the ITO thickness of 13 nm and 18 nm. However, in the case of ITO thickness of Figure 4f, 22 nm, we could not find the optimized points on the map and this implies that the 22 nm thickness of the ITO layer cannot make the target optical performance even if we change the CPL and the cathode thickness. Thus, the additional ITO thickness was not considered for the calculation conditions for achieving the target optical performance. Therefore, we can conclude that optimized points that provide excellent color performance can be found within the thickness of 13 nm and 21 nm ITO.

**Figure 5:**
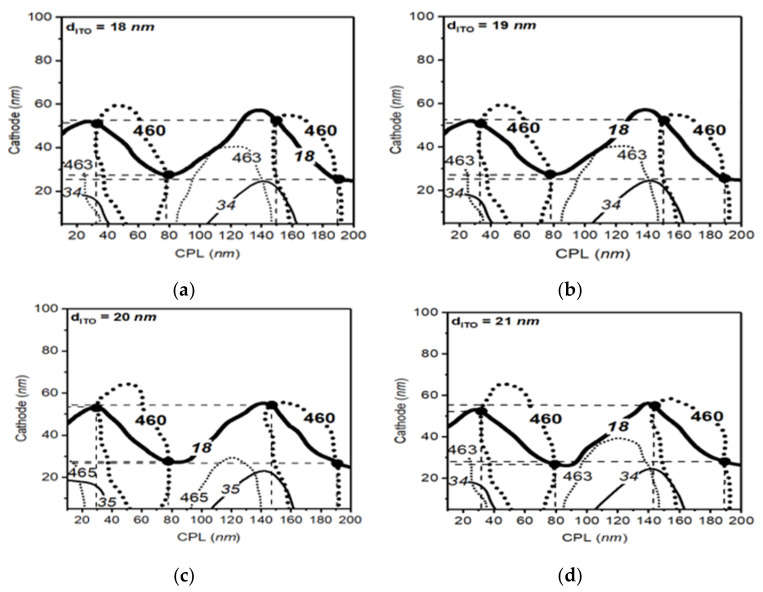
The parameter space result of blue structure with ITO thickness from 18 to 21 nm. Solid lines and dotted lines in the figure show the calculated output lines in each optical thickness of the CPL and cathode layer. Bold lines in the figure represent the desired optical conditions with the peak wavelength of 460 nm and bandwidth of 18 nm for the blue OLED (**a**–**d**).

**Figure 6:**
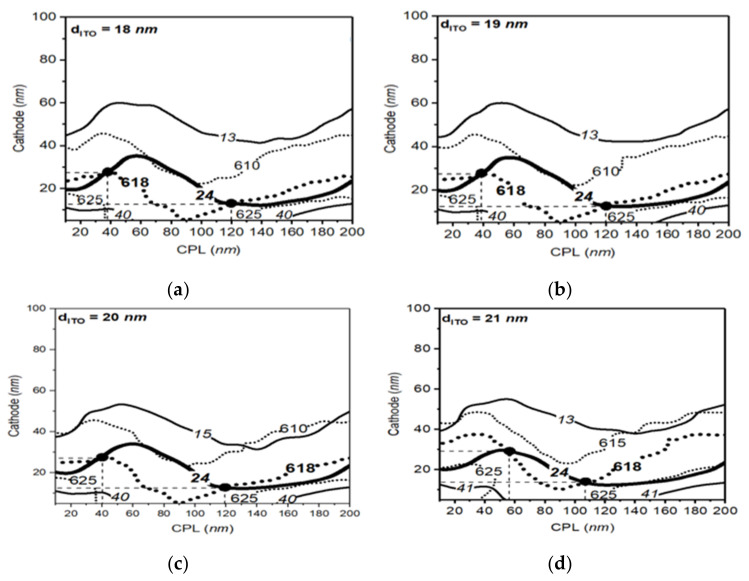
The parameter space result of red structure with ITO thickness from 18 to 21 nm. Solid lines and dotted lines in the figure represent the optical conditions that can provide the calculated bandwidth and the peak wavelength, respectively. Bold lines in the figure represent the desired optical conditions with the peak wavelength of 618 nm and bandwidth of 24 nm for the red OLED (**a**–**d**).

In Figure 5 and Figure 6, we applied the parameter space methods to blue and red OLED structures in same way to the case of the green OLED cell to show optimized thickness conditions corresponding to peak wavelengths and bandwidths of 460 nm and 18 nm, and 618 nm and 24 nm, respectively. In the case of the blue OLED cell, we found four optimized conditions between the ITO thickness of 18 nm and 21 nm, as shown in Figure 5 on the map. The red OLED cell shows two optimized points between the ITO thickness of 18 nm and 21 nm, as shown in Figure 6. Thus, we can confirm that the optimized points of the long wavelength OLED device are reduced compared to the case in the short wavelength OLED device.

From the above calculated parameter space maps, we were able to design each optical length of the inorganic layers for R, G, and B OLED cells. For a practical approach in the full colored OLED cell, however, we needed to apply same optical thickness of the inorganic layers in the cell. To find out which points were optimized for the desired three colors, therefore, we inspected the optical optimized points of three R, G, and B OLED cells as functions of the optical thickness of the ITO, the Cathode, and the CPL on the parameter space map. In the calculation, the permitted deviations of the expected peak wavelength and bandwidth were ±1 nm and ±2 nm, respectively.

**Figure 7:**
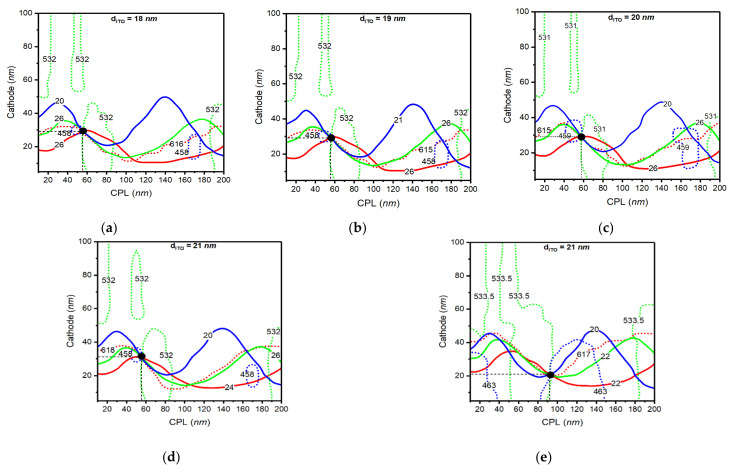
Calculated parameter space for the Red, Green, and Blue organic light-emitting diode (OLED) cell. (**a**–**e**) Optimized points for three colors with smallest error at from the indium tin oxide (ITO) thickness of 18 nm to 21 nm. Solid lines and dotted lines in the figure show the calculated output lines in each optical thickness of the capping layer (CPL) and cathode layer.

**Figure 8:**
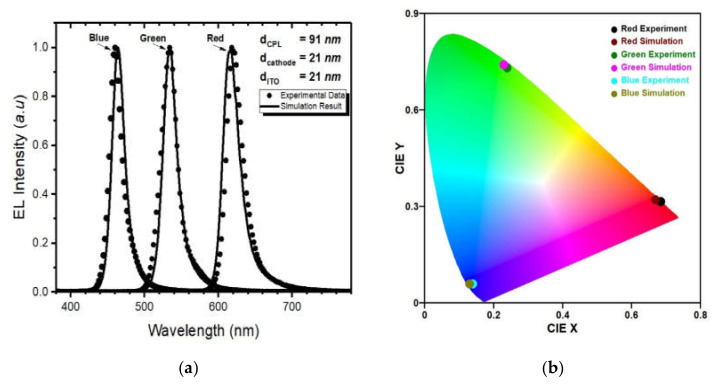
(**a**) Comparison of the measured optical spectrum and the calculated spectrum of the red, green, blue OLED cell and (**b**) CIE coordinates at the CPL thickness of 91 nm, the cathode thickness of 21 nm, ITO thickness of 21 nm.

Figure 7 shows the calculated points optimized for the R, G, and B color OLED cell on the maps. We found the optimized points within the ITO thickness of 18 to 21 nm. In the Figure 7, the ITO thickness between 18 and 20 nm permits a single optimized optical condition, and we found two optimized conditions in the case of the ITO thickness of 21 nm. From the Figure 7a–e, the optimized points within the deviation value from the target optical performances for all three colors were obtained with CPL (55 nm)/cathode (29 nm) at ITO thicknesses of 18 nm and 19 nm, with CPL (58 nm)/cathode (29 nm) at ITO thickness of 20 nm, with CPL (55 nm)/cathode (31 nm) and CPL (91 nm)/cathode (21 nm) at ITO thickness of 21 nm. Therefore, we found the optical condition that satisfied all the colored OLED cells at the CPL thickness of 91 nm, the cathode thickness of 21 nm, and the ITO thickness of 21 nm, respectively. For verification, we measured experimental results of the emitting spectrum from the R, G, and B OLED cell with calculated optical thicknesses, as shown in Figure 8. As shown in Figure 8a, we were able to observe that the measured spectrum and the calculated spectrum show small differences regarding the peak wavelength and the bandwidth, which are about 3 nm and 2 nm, respectively. We assumed that the small difference in terms of the bandwidth and the peak wavelength may have occurred because of the evaporation errors of each layer thickness in the process. From the measured spectrum of the OLED cell, we could also calculate color coordinate values. Figure 8b shows comparison of the measured color positions of the R, G, and B OLED cell with the calculated color values in the color coordinate system. In Figure 8b, the measured color values of the R, G, and B spectrum were (0.67, 0,32), (0.23, 0.74), and (0.13, 0.06), respectively, and the results show well-matched performances in all three colors.

## 4. Conclusions

We applied the parameter space map to an RGB OLED and obtained optimized inorganic layer thicknesses for the desired color performances of all three colors. On the map, we found that the expected peak wavelengths and the bandwidths of the three R, G, and B colors can be obtained with almost the same thicknesses of CPL, cathode, and ITO at single optimized points. In order to verify the calculation, we compared the simulated spectra and the experimental data of the R, G, and B OLED cell and found well-matched performances for all three colors. As a result, the expected optical performance was attained for all three colors: red, green, and blue in the single OLED structure This implies that the parameter space method that we proposed can show the effect of organic and inorganic layers on the electro-optical characteristics at a glance and directions on how to optimize the optical path length of each layer for achieving the desired properties.

## Figures and Tables

**Table 1:** Optical properties of the uniaxial materials in the organic light-emitting diode (OLED) structure.

Layers	Thickness	Refractive $\mathrm{Index}\;(n_{z})$	Extinction $\mathrm{Coefficient}\;(k_{z})$	Refractive $\mathrm{Index}\;(n_{x},n_{y})$	Extinction $\mathrm{Coefficient}\;(k_{x},k_{y})$
HTL	93 nm	1.85505	0	1.63631	0
R Prime	68 nm	1.86839	$1.00\;\times \;10^{-5}$	1.67377	0.00181
*p*HTL	9 nm	1.86117	0	1.65279	0

**Table 2:** Optical properties of the isotropic materials in the OLED structure.

Layers	Thickness	Refractive Index	Extinction Coefficient
CPL	Varied	1.92392	0
Cathode	Varied	1.2068	0
Cathode 1	3	0.175646	2.76587
ETL	30	1.84152	$7.36\;\times \;10^{-4}$
R-EML	15	2.04304	$1.09\;\times \;10^{-4}$
G-EML	15	1.95648	$1.41\;\times \;10^{-3}$
B-EML	15	1.84815	0
EBL	15	1.81139	$1.90\;\times \;10^{-5}$
G Prime	40	1.97543	0
ITO	Varied	2.0227	$2.23\;\times \;10^{-2}$
Anode	1000	0.14579	3.2904

## Data Availability

Not Applicable.
